# Investigating the effect of progressive truncations at the ALS-linked protein TDP-43 RRM2 on its aggregation mechanism

**DOI:** 10.3389/fmolb.2026.1849627

**Published:** 2026-06-24

**Authors:** Greta Grassmann, Matteo Amadei, Arianna Lardieri, Linda Celeste Montemiglio, Alessandra Anna Passeri, Maurizio Mattarelli, Lorenzo Di Rienzo, Mattia Miotto, Giancarlo Ruocco, Edoardo Milanetti

**Affiliations:** 1 Center for Life Nano and Neuro Science, Italian Institute of Technology, Rome, Italy; 2 Institute of Molecular Biology and Pathology c/o Department of Biochemical Sciences “Alessandro Rossi Fanelli”, Sapienza, University of Rome, National Research Council, Rome, Italy; 3 Department of Biochemical Sciences “Alessandro Rossi Fanelli”, Sapienza, University of Rome, Rome, Italy; 4 Department of Physics and Geology, University of Perugia, Perugia, Italy; 5 Department of Physics, Sapienza, University of Rome, Rome, Italy; 6 Department of Human Sciences, Link Campus University, Rome, Italy

**Keywords:** aggregation model, ALS (Amyotrophic lateral sclerosis), molecular dynamics simulation, protein aggragation, protein structure and function, TDP43

## Abstract

Amyotrophic lateral sclerosis is a neurodegenerative disease characterized by inclusions of TDP-43 protein. C-terminal fragments (CTFs) of TDP-43, generated by cleavage within its second RNA recognition motif (RRM2), have been found forming aggregates in patients. Aggregation has often been attributed to the C-terminal domain, but increasing evidence indicates that RRM2 fragments contribute to pathological inclusions. We performed extensive molecular dynamics simulations to investigate the changes resulting from the truncation that could lead to aggregation. We analyzed the full RRM2 domain (fRRM2, residues 192–261) and two fragments commonly observed in CTFs (tRRM2A, residues 220–261, and tRRM2B, residues 209–261). We found that truncation results in distinct aggregation-prone states. tRRM2B appears to rely on β -sheet elements associated with amyloid-like aggregation, whereas tRRM2A exhibits higher structural variability and a reduced β -content, suggesting a phase separation-like aggregation mechanism. We further simulated an extended fragment of tRRM2A, tRRM2A-l (residues 220–269). Although its predicted aggregation propensity remains largely unchanged, tRRM2A-l exhibits increased structural flexibility, and a stronger exposure of Nuclear Export Signal residues. Our results indicate that subtle differences in RRM2 fragment length influence potential misfolding pathways. Future studies and therapeutic strategies to prevent TDP-43 aggregation should carefully consider the specific domain adopted.

## 1 Introduction

Amyotrophic lateral sclerosis (ALS) is one of the most common adult degenerative motor neuron diseases, affecting approximately two individuals per 100,000 each year and typically leading to death within 5 years of onset (Boillée et al., 2006; Pasinelli and Brown, 2006). About 90% of ALS cases are sporadic (Pasinelli and Brown, 2006; Neumann et al., 2006; Arai et al., 2006) and are characterized by the presence of cytoplasmic inclusions containing the Transactive response DNA binding protein 43 kDa (TDP-43). Notably, TDP-43 aggregation is also implicated in other neurodegenerative disorders, including Frontotemporal Dementia (FTD) and Alzheimer’s Disease (AD) (Arai et al., 2006; Zuo et al., 2021; Jo et al., 2020). Understanding how such protein inclusions form is essential for elucidating the molecular basis of aggregate-related neurodegeneration and may inform therapeutic strategies (Baloh, 2011). However, despite extensive study, the precise molecular mechanisms underlying TDP-43 misfolding and aggregation remain incompletely understood (Pasinelli and Brown, 2006; Kiernan et al., 2011).

TDP-43 is a 414-amino-acid RNA-binding protein composed of two RNA recognition motifs (RRM1 and RRM2) and intrinsically disordered N- and C-terminal domains (NTD and CTD) (Jiang et al., 2017). The RRM2 domain includes the Nuclear Export Signal (NES), which increases the transport to the cytoplasm and serves as a molecular hazard linking physiological folding with pathological misfolding and aggregation. In physiological conditions, NTD-mediated head-to-tail oligomerization spatially separates the aggregation-prone CTDs of individual monomers, thus preventing pathological aggregation (Afroz et al., 2017). During neurodegenerative diseases, TDP-43 undergoes a wide array of post-translational modifications, including phosphorylation, acetylation, ubiquitination, oxidation, and cleavage (François-Moutal et al., 2019). C-terminal fragments (CTFs) resulting from cleavage in the RRM2 domain have an increased aggregation propensity compared to full-length TDP-43 (Wang et al., 2013; Mackness et al., 2014; Testi et al., 2026), and have been found in cytoplasmic aggregates from ALS patient tissues (Jiang et al., 2017; Afroz et al., 2017; Tavella et al., 2018; Prasad et al., 2019). Two major classes of CTFs have been reported, depending on the RRM2 cleavage site: one truncated at residue 208 (residues 209–414) and another truncated at residue 219 (residues 220–414) (Guenther et al., 2018; Buratti, 2018).

Although aggregation is often attributed primarily to the CTD, it has been shown that the RRM2 domain itself contributes critically to the aggregation process (Johnson et al., 2008). RRM2 forms the stability core of full-length TDP-43 (Mackness et al., 2014; Kuo et al., 2009), yet its isolated fragments display a strong tendency to aggregate due to the loss of stabilizing interdomain interactions with RRM1 (Mackness et al., 2014). Cleavage within RRM2 also exposes its aggregation-prone β -strands (Wang et al., 2013; Kumar et al., 2019). These strands are buried in the native state, but can form fibrils *in vitro* (Tavella et al., 2018).

Remarkably, RRM2 fragments can aggregate even in the absence of the CTD (Wang et al., 2013; Mackness et al., 2014), highlighting their intrinsic propensity for misfolding. These finding suggest that investigating the structure and dynamical variations of the truncated RRM2 fragments is important for understanding what drives the formation of large aggregates found in neurodegenerative diseases such as ALS.

**FIGURE 1:**
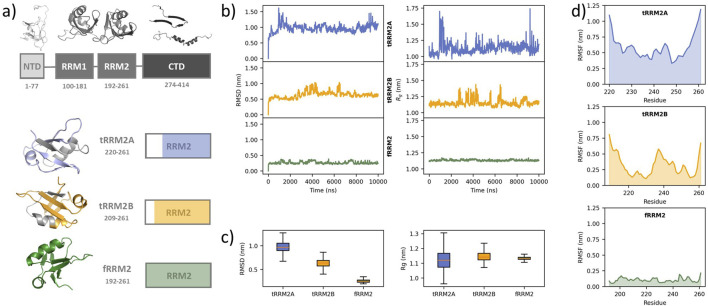
MD characterization of the RRM2 domain and its truncated fragments. **(a)** On top, schematic representation of the full-length TDP-43 protein. TDP-43 comprises an NTD, two RRM domains, and a disordered C-terminal domain. As examples, the cartoon representation of the structure of an NTD portion [PDB ID: 2N4P (Mompeán et al., 2016)] is shown in light grey, the structure of the tandem RRM1-RRM2 domains [PDB ID: 4BS2 (Lukavsky et al., 2013)] in gray. Two fragments of the CTD are reported in dark gray, counting residues 288–319 [PDB ID: 6N3C (Cao et al., 2019)] and 311–360 [PDB ID: 2N3X (Jiang et al., 2017)]. On bottom, the three RRM2-derived constructs analyzed: fRRM2 (residues 192–261), tRRM2B (209–261), and tRRM2A (220–261). The cartoon representation of the RRM2 structure (from PDB ID: 4BS2) is shown in green for fRRM2, while the truncated fragments corresponding to those identified in CTFs are shown in orange (tRRM2B) and blue (tRRM2A). **(b)** Time evolution of the RMSD (left) and Rg (right) with respect to the starting structure over 10μs of MD simulations. From top to bottom: tRRM2A, tRRM2B, and fRRM2. **(c)** Distribution of RMSD (left) and Rg (right) values from b) represented as boxplots for fRRM2 and its two truncated variants. **(d)** Per-residue RMSF profiles for tRRM2A, tRRM2B, and fRRM2 (top to bottom).

Because of the high aggregation propensity of truncated RRM2 (Furukawa et al., 2011), these systems are difficult to investigate experimentally. In silico techniques are an essential tool to characterize their structure and dynamics at the atomic level. To this end, we performed 10 μ s-long MD simulations of full-length RRM2 (fRRM2, residues 192–261) and of two disease-relevant truncated fragments, tRRM2A and tRRM2B, corresponding to residues 220–261 and 209–261, respectively (Figure 1a). Our objective was to characterize how truncation affects the conformational landscape, stability, and aggregation propensity of RRM2-derived fragments, thereby shedding light on early misfolding events that may precede aggregation.

To further characterize the conformational variability and aggregation-prone regions sampled by RRM2 fragments, we introduced an extended construct, tRRM2A-l, encompassing residues 220–269. In the context of ALS, both RRM2 constructs extending to residue 261 and to residue 269 have been investigated in previous studies (Morgan et al., 2017; Grassmann et al., 2021): by comparing tRRM2A-l structural dynamics with those of tRRM2A, we assessed how the inclusion of additional residues at the C termini end influence conformational stability and the tendency to aggregate of RRM2 fragments. This kind of observations can help to better understand the molecular mechanisms underlying CTFs aggregation and thus pave the way for the development of therapeutics.

## 2 Results

To assess how different cleavages affect the structure and stability of the RRM2 portion in CTFs, we performed 10 μ s-long MD simulations of fRRM2 (192-261), tRRM2A (220-261), and tRRM2B (209-261), and analyzed their conformational dynamics and overall stability. Observing their different behavior, we hypothesized that the two fragments could follow different aggregation models.

Since most *in vitro* and *in silico* analyses study RRM2 fragments terminating at residue 261 or 269, we also tested the dynamic and structural variations that residues at the CTD-end can cause. Specifically, we simulated and analyzed the trajectory of tRRM2A-l, which contains eight additional residues at the C-terminus compared with tRRM2A. We found that these residues induce conformational variation in tRRM2A-l compared to tRRM2A, enabling the unfolding of this fragment.

### 2.1 RRM2 cleavage results in higher structural instability

A preliminary analysis of the MD simulations of full-length RRM2 (fRRM2) and its two truncated fragments (tRRM2A and tRRM2B), shown in Figures 1b,c, indicates that fRRM2 is the most structurally stable construct. Its backbone Root Mean Square Deviation (RMSD) remains low and steady throughout the trajectory, with an average value of (0.26 ± 0.04) nm. As expected, the truncated fragments exhibit larger conformational fluctuations, with mean RMSD values of (0.60 ± 0.11) nm for tRRM2B and (0.97 ± 0.12) nm for tRRM2A.

A similar trend is observed for the radius of gyration $\left(R_{g}\right)$ . fRRM2 shows the lowest variability (standard deviation of 0.01 nm), whereas tRRM2A displays the largest (standard deviation of 0.09 nm). Despite these differences in flexibility, the mean $R_{g}$ values are comparable across all constructs (1.13, 1.15, and 1.13 nm for fRRM2, tRRM2B, and tRRM2A, respectively), suggesting that the overall compactness is largely preserved even in the truncated forms. The statistically distinct RMSD values, however, highlight the greater structural instability of the shorter fragments.

To further characterize these differences, we computed the Root Mean Square Fluctuation (RMSF) for each residue, which reflects the average positional deviation from its mean coordinates over time and provides a residue-level measure of local flexibility. The RMSF profiles (Figure 1d) reveal pronounced variations among the three constructs. Both truncated forms show higher fluctuations, particularly at the termini, consistent with enhanced mobility due to the loss of stabilizing contacts present in the full-length domain. tRRM2A exhibits the largest overall fluctuations (up to ∼ 1 nm), indicative of marked structural instability, whereas tRRM2B displays intermediate flexibility with peaks around residues 235–245 ( ∼ 0.4 nm), suggesting partial stabilization but reduced global rigidity. In contrast, fRRM2 maintains consistently low RMSF values ( < 0.2 nm) across the entire sequence, confirming its stability and well-folded conformation.

Overall, these results confirm that progressive truncation of the RRM2 domain increases local flexibility and decreases structural stability, while the full-length construct remains compact and robust throughout the simulation.

### 2.2 Progressive RRM2 truncations increasingly destabilize secondary structures and inter-molecular core contacts

**FIGURE 2:**
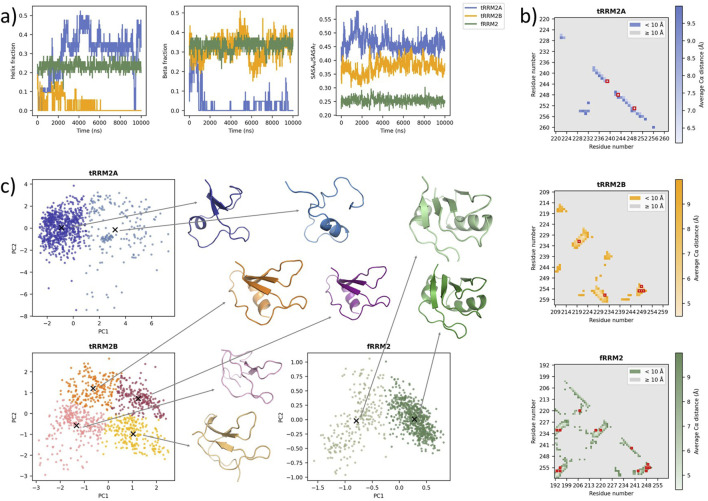
Structural and dynamic analysis of the RRM2 domain and its truncated fragments. **(a)** Time evolution of the fraction of α-helical residues, β-strand residues, and the solvent-accessible surface area ratio (SASAH/SASAT) for tRRM2A (blue), tRRM2B (orange), and fRRM2 (green). **(b)** Average Cα–α distance maps computed over the MD trajectories for non-neighboring residues (i.e., separated by at least two positions along the sequence). Only residue pairs with an average distance below 10° are shown, using blue, orange, and green color scales for tRRM2A, tRRM2B, and fRRM2, respectively. Contacts between ILV residues (i.e. Cα–α distances lower than 8°) are marked by red squares. **(c)** Two-dimensional projections of the sampled conformations onto the subspace spanned by the first two PCs of the covariance matrix of atomic displacements visited during the simulation of the two fragments and the full RRM2. A k-means clustering analysis was performed on each trajectory. Points are colored according to their assigned cluster, and cluster centroids are marked with crosses. The representative structures corresponding to the centroids are shown as cartoon models in the respective colors.

To further investigate the structural consequences of the RRM2 cleavages, we analyzed the variations in secondary and tertiary structure composition throughout the simulations. Specifically, we monitored the time evolution of the fraction of helical residues, strand residues, and the ratio between the solvent-accessible surface area (SASA) of hydrophobic residues ( ${\text{SASA}}_{H}$ ) and the total SASA ( ${\text{SASA}}_{T}$ ). As shown in Figure 2a, fRRM2 maintains a stable secondary structure composition over the entire simulation, with nearly constant fractions of α -helix and β -strand content. In contrast, tRRM2A and tRRM2B exhibit pronounced temporal fluctuations, reflecting partial unfolding events and rearrangements of secondary structure elements. These variations suggest a disruption of the native fold and an increased exposure of hydrophobic residues normally buried within the compact core of fRRM2, confirmed by their higher values of ${\text{SASA}}_{H}/{\text{SASA}}_{T}$ compared to fRRM2. Such exposure may facilitate nonspecific intermolecular interactions, which often represent the initial steps of aggregation. Conversely, fRRM2 retains a stable β -rich core and a consistent secondary structure content, which likely protects it from aggregation by preserving a well-defined tertiary packing.

This observation is supported by the average C α –C α contact maps reported in Figure 2b, which show inter-residue distances between non-neighboring residues (i.e., separated by at least two residues along the chain). fRRM2 displays a well-defined and dense contact network. Several of the contacts are between isoleucine (I), leucine (L), and valine (V) residues: the highly interconnected hydrophobic cluster formed by these residues in the core of RRM2 is known to confer hight stability to this domain (Mackness et al., 2014). Several of these contacts provide a link between the N- and C-termini of fRRM2 (V193/ILE257, VAL195/ILE257). On the other hand, both truncated fragments show a marked loss of long-range interactions and ILV contacts, consistent with increased flexibility and structural disorder.

Principal Component Analysis (PCA) of the atomic coordinates sampled during the MD simulations (Figure 2c) further corroborates this trend. Projection of the MD trajectories onto the two PCs provides an essential representation of the dominant motions sampled during the simulations. fRRM2 explores a narrow conformational subspace, indicative of structural stability, whereas tRRM2B and, more prominently, tRRM2A sample broader and more heterogeneous regions of the conformational landscape, consistent with enhanced conformational plasticity and partial unfolding. To characterize the most populated conformational states of each fragment and provide a comprehensive description of their aggregation propensity across different structural arrangements, we performed a clustering analysis of the PCA projections. This approach enabled the identification of the most representative conformations for each construct, defined as those closest to the centroids of the sampled conformational basins. The PCA projections of fRRM2 were grouped into two clusters, accounting for 78% and 22% of the data points respectively (dark and light blue in Figure 2c). Four clusters were identified for tRRM2B, with relatively similar populations ranging from 21% to 30%. A more heterogeneous distribution of cluster sizes was observed for fRRM2, where the largest cluster (dark green in Figure 2c) comprises 73% of the points. Since each identified cluster contained at least one fifth of the total data points, we proceeded to analyze the representative conformation of each cluster. In fRRM2, the first conformation includes four β -strands - β 1 (193–196), β 2 (218–221), β 3 (229–234), and β 4 (256–259)- and two α -helices - α 1 (204–214) and α 2 (237–245)-. The second conformation retains a similar organization, with minor variations at the termini of β 1, β 3, β 4, and α 2. The tRRM2B truncation leads to the loss of β 1 and a substantial rearrangement of the remaining secondary elements. The first conformation has no α -helices and small variation in the three remaining β -sheets: β 2 (219-221), β 3 (229-235), and β 4 (256-258). Compared to fRRM2, an additional sheet is formed, $\beta 3_{\mathrm{bis}}$ (237-239). The second conformation has no secondary structures. The third conformation has β 2, β 3, and β 4. It also present an α -helix, including residues from 210 to 217. The fourth conformation has no α helices and two β -sheets including residue ranges 219-221 and 231-233. This partial loss of secondary structure is consistent with circular dichroism (CD) measurements (Mackness et al., 2014).

The two most populated conformations of tRRM2A are characterized by even greater structural disruption. The first retains only two short β -sheets (220–223 and 257–260) and a single α -helix (237–241), whereas the second maintains one α -helix involving residues 246–253, with the rest of the structure largely disordered.

Overall, these results demonstrate that progressive truncation of the RRM2 domain destabilizes both its secondary and tertiary structure, leading to increased conformational variability, reduced intramolecular cohesion, and enhanced exposure of aggregation-prone regions.

### 2.3 RRM2 fragments follow different aggregation models

Defining the conformational landscape explored by protein fragments and characterizing their representative conformations through MD simulations provide a fundamental framework for investigating how they can self-interact and undergo aggregation. Numerous computational tools have been developed to predict protein aggregation propensities (Pallarès and Ventura, 2019), several of which explicitly account for protein dynamic fluctuations (Navarro and Ventura, 2022). This highlights the importance of approaches capable of capturing conformational variability when assessing aggregation behavior. Among these methods, one of the most widely used tools for aggregation prediction is the Aggrescan3D (A3D) 2.0 server (Kuriata et al., 2019). A3D performs structure-based aggregation predictions by integrating three-dimensional structural information with conformational fluctuations, modeled through the CABS-flex approach (Jamroz et al., 2013). The method identifies solvent-exposed aggregation-prone regions and assigns a residue-specific aggregation score (A3D score) that depends on both the amino acid sequence and the local structural environment.

We applied A3D to the representative conformations obtained from the MD simulations of fRRM2, tRRM2A, and tRRM2B in order to further investigate how RRM2 cleavage affects the dynamic properties and aggregation propensity of the resulting fragments.

**FIGURE 3:**
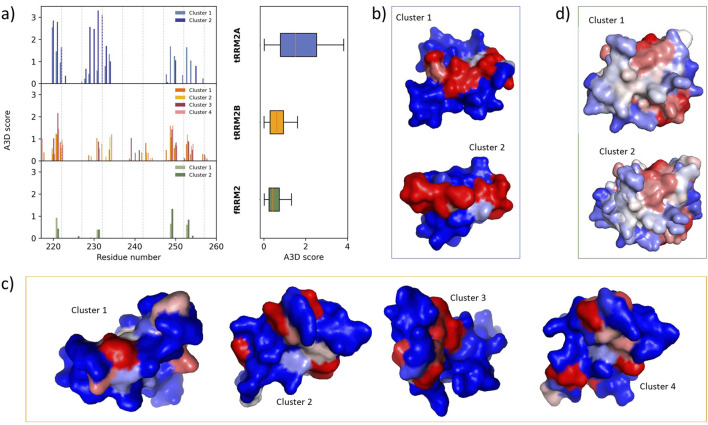
Aggregation propensity of the representative conformations of the full RRM2 and its fragments, as computed with the A3D server. **(a)** Per-residue A3D scores calculated for each representative conformation. Results for fRRM2, tRRM2B, and tRRM2A are shown in green, orange, and blue, respectively. On the right, boxplots summarize the distribution of A3D scores across all conformations for each construct. **(b)** Molecular surfaces of the two representative conformations identified for tRRM2A. Residues are colored according to their A3D scores, with red indicating higher aggregation propensity. **(c)** Same as in **(b)**, but for the tRRM2B fragment. **(d)** Same as in **(b)**, but for the full-length fRRM2 construct.

Figure 3a reports the per-residue A3D scores for the representative conformations identified for the full RRM2 domain and its truncated fragments.

Figures 3b–d display the corresponding molecular surfaces, colored according to each residue’s A3D score, from blue (soluble) to red (aggregation-prone). As expected, fRRM2 exhibits the lowest aggregation propensity, with only a few residues showing positive A3D values, all below ∼ 1.3. In contrast, tRRM2B conformations contain a higher number of residues with positive A3D scores, while tRRM2A displays the strongest aggregation tendency according to A3D, with values reaching up to ∼ 4.

These results suggest that the two fragments may aggregate through distinct mechanisms. (Prasad et al., 2019; Guenther et al., 2018). Tables 1–3 list all residues predicted by A3D as aggregation-prone in each representative conformation, highlighting those located within β -strands. Notably, in tRRM2B, β -sheet residues, already shown in Figure 2a) to be abundant in this fragment, are frequently identified as aggregation hotspots. This observation is consistent with the formation of β -mediated interfaces and supports a β -sheet–driven aggregation mechanism for tRRM2B, in line with previous computational results showing that tRRM2B dimers formed through β -sheet pairing are both more likely to form and more stable than alternative interactions (Grassmann et al., 2021). Conversely, in tRRM2A, aggregation-prone residues are less frequently located within β -sheets. This fragment is, however, included in CTFs that are experimentally known to be highly aggregation-prone and neurotoxic in ALS, though the underlying mechanism remains unclear (Elena Cicardi et al., 2018; Wang et al., 2015; Chen et al., 2016). Experimental studies have shown that CTFs encompassing tRRM2A form non-fibrillar gel-like inclusions (Riemenschneider et al., 2022), suggesting a different aggregation pathway involving weak, multivalent interactions and phase-separation–like behavior (Gomes and Shorter, 2019; Martin and Mittag, 2018). The high A3D scores observed for tRRM2A residues thus support the hypothesis that this shorter fragment contributes to TDP-43 CTF aggregation through liquid-like phase separation (LLPS)-mediated mechanisms, potentially leading to the formation of gel-like or amorphous aggregates rather than amyloid fibrils.

**TABLE 1:** A3D scores for tRRM2A residues. For each representative conformation of tRRM2A, the Table lists the residues predicted to be aggregation-prone along with their corresponding A3D scores. Residues participating in β -sheet structures are highlighted in red.

Cluster 1		Cluster 2	
Residue	A3D score	Residue	A3D score
VAL220	2.5427	VAL220	2.8561
PHE221	1.4575	PHE221	2.7945
ILE222	0.9519	ILE222	1.6318
ALA228	0.2168	PRO223	0.3525
PHE229	0.4368	ARG227	0.1062
PHE231	0.59	ALA228	0.6728
THR233	0.7839	PHE229	2.5634
PHE234	1.3283	ALA230	2.4771
LEU248	0.4234	PHE231	3.3055
ILE249	1.684	VAL232	3.1166
ILE250	1.2467	THR233	1.707
GLY252	0.3797	PHE234	1.0056
ILE253	1.6415	LEU248	0.0555
SER254	1.0459	ILE250	1.0501
ILE257	0.2356	VAL255	0.8058

**TABLE 2:** A3D scores for tRRM2B residues. For each representative conformation of tRRM2B, the Table lists the residues predicted to be aggregation-prone along with their corresponding A3D scores. Residues participating in β -sheet structures are highlighted in red.

Cluster 1		Cluster 2		Cluster 3		Cluster 4	
Residue	A3D score	Residue	A3D score	Residue	A3D score	Residue	A3D score
PHE210	0.6538	PHE211	1.009	VAL220	1.0203	PHE211	1.613
PHE211	0.203	SER212	0.0116	PHE221	2.1597	SER212	0.7335
VAL217	0.9398	TYR214	0.5034	ILE222	0.9426	TYR214	0.6514
MET218	0.3793	VAL217	0.0127	PHE231	0.8739	GLY215	0.1594
VAL220	0.5636	VAL220	0.2975	ILE239	1.0346	VAL217	0.9982
PHE221	1.2319	PHE221	1.1838	ALA240	0.3696	VAL220	0.31
ILE222	0.8438	ILE222	0.5839	GLN241	0.4824	PHE221	1.4497
PHE229	0.2587	VAL232	0.7678	ILE249	1.4242	ILE222	0.7337
PHE231	1.0396	THR233	0.1918	ILE250	0.8294	PHE229	0.2006
PHE234	0.5035	PHE234	1.0769	GLY252	0.1365	PHE231	0.5583
ILE239	0.1043	LEU243	0.3644	ILE253	0.8912	PHE234	1.1997
SER242	0.384	CYS244	0.126	SER254	0.502	LEU243	0.3694
LEU243	0.8019	ILE249	1.0711	ILE257	0.2814	CYS244	0.1435
LEU248	0.4915	ILE250	0.7252			LEU248	0.0475
ILE249	1.5842	GLY252	0.0332			ILE249	1.5968
ILE250	0.5745	ILE253	1.1914			ILE250	0.6672
ILE253	0.7532	SER254	0.6723			GLY252	0.0902
SER254	0.3082					ILE253	0.8511
ILE257	0.3012					SER254	0.7637
SER258	0.1127					ILE257	0.2068

**TABLE 3:** A3D scores for fRRM2 residues. For each representative conformation of fRRM2, the Table lists the residues predicted to be aggregation-prone along with their corresponding A3D scores. Residues participating in β -sheet structures are highlighted in red.

Cluster 1		Cluster 2	
Residue	A3D score	Residue	A3D score
PHE221	0.9264	PHE221	0.4257
PHE231	0.385	PHE226	0.0884
ILE249	0.6523	PHE229	0.0215
ILE253	0.6049	PHE231	0.3931
		ILE249	1.3271
		ILE253	0.8299
		SER254	0.1097

### 2.4 Minor sequence variations can induce substantial alterations in the conformational landscape and aggregation pathway of RRM2 fragments

To further investigate the aggregation propensity of the fragments associated to higher A3D scores, tRRM2A, we considered an alternative RRM2 domain definition commonly reported in literature, in which residues up to the 269th are associated to RRM2 (Lukavsky et al., 2013). This led to the design of an extended fragment, tRRM2A-l, which contains eight additional residues toward the CTD compared to tRRM2A. To characterize the structural behavior of this extended fragment, we conducted a 10 μ s-long MD simulation.

**FIGURE 4:**
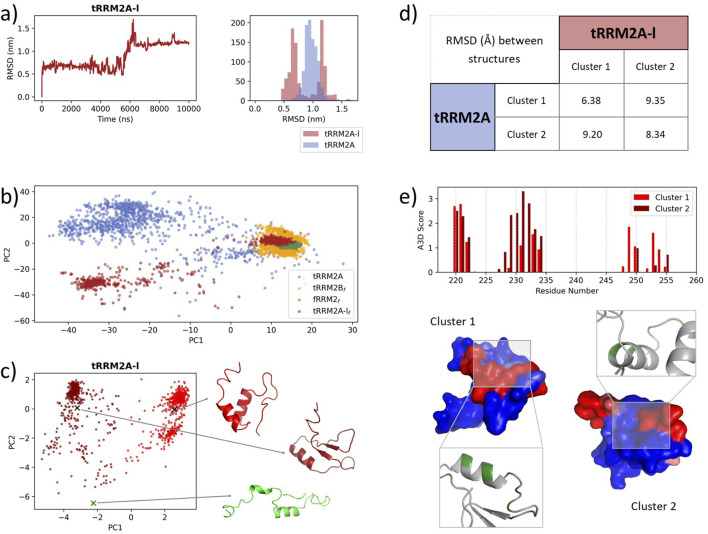
MD characterization of the extended tRRM2A-l fragment and its aggregation propensity. **(a)** On the left, time evolution of the RMSD of tRRM2A-l relative to its starting structure over 10 μs of simulation. On the right, RMSD distributions for tRRM2A-l (red) and tRRM2A (blue) (see Figure 1b). **(b)** Two-dimensional projection of the conformations sampled during the simulations onto the subspace spanned by the first two PCs derived from the covariance matrix of atomic displacements of the common residues. The analysis includes the three fragments and the full RRM2 domain. Points are colored according to the corresponding construct, as indicated in the legend. **(c)** Two-dimensional projection of the sampled conformations onto the subspace defined by the first two PCs of the covariance matrix of atomic positions during the tRRM2A-l simulation. K-means clustering was performed, and points are colored according to cluster assignment. Cluster centroids are marked with crosses, and cartoon representations of the corresponding conformations are shown. A green cross highlights the projection of the most unfolded conformation, extracted from the simulation frame for which the highest RMSD was computed as shown in a). The corresponding molecular structure is represented in green. **(d)** RMSD values computed between the tRRM2A-l representative conformations identified in panel b and the representative conformations of tRRM2A shown in Figure 2c. **(e)** A3D-predicted residue aggregation propensities for each representative conformation. Molecular surfaces are shown below, with residues colored according to their A3D scores. In the zoomed molecular portions, NES residues are highlighted in green for both conformations.

The time evolution of the RMSD, shown in Figure 4a, reveals a pronounced peak around 6500 ns, indicating the transient formation of an unstructured conformation. Following this event, the fragment rapidly equilibrates into a distinct structural state. In contrast to tRRM2A, whose RMSD distribution is narrowly centered around ∼ 1 nm, tRRM2A-l exhibits a bimodal RMSD distribution with mean values of (0.66 ± 0.07) nm and (1.17 ± 0.05) nm, reflecting the presence of two dominant conformational states.

To compare the effect of the different truncations on the RRM2 domain, we performed a PCA of the MD atomic fluctuations focusing on the region common to all constructs (residues 220-261), hereafter referred to as tRRM2Af, tRRM2Bf, fRRM2f, and tRRM2A-lf. As shown in Figure 4b, analysis of this shared segment confirms the trends observed for the full systems: fRRM2f exhibits the lowest structural variability, whereas progressively shorter constructs sample increasingly broader regions of the conformational space. Notably, both tRRM2Af and tRRM2f-lf populate two distinct regions. One overlaps with the conformational space sampled by fRRM2f and tRRM2Bf, while the other is shifted along both PC1 and PC2 and differs between the two fragments.

To further characterize this behavior, we analyzed the conformational ensemble of tRRM2A-lf in greater detail. PCA of its MD atomic fluctuations, combined with clustering analysis, reveals two well-separated regions with comparable populations (55% and 44% of the data points, shown in red and dark red in Figure 4c), supporting the occurrence of a major structural transition. The representative conformation of the first cluster retains two β-strands (residues 229-231 and 256-258) and preserves the α3 helix. In the second conformation, unfolding leads to the loss of these β-sheets and the formation of a new α-helix spanning residues 251-259.

As illustrated in Figure 4d, both conformations are clearly distinct from those adopted by the tRRM2A fragment.

Despite these structural differences, the predicted aggregation propensities from the A3D server (Figure 4e; Table 4) remain comparable between tRRM2A and tRRM2A-l. Nonetheless, the pronounced conformational changes induced by extending the CTD boundary suggest that further investigation of potential fold–unfold transitions is warranted.

**TABLE 4:** A3D scores for tRRM2A-l residues. For each representative conformation of fRRM2A-l, the Table lists the residues predicted to be aggregation-prone along with their corresponding A3D scores. Residues participating in β -sheet structures are highlighted in red.

Cluster 1		Cluster 2	
Residue	A3D score	Residue	A3D score
VAL220	2.696	VAL220	2.4979
PHE221	2.7799	PHE221	2.2868
ILE222	1.2315	ILE222	1.4275
PHE229	0.1773	PRO223	0.0233
PHE231	1.0949	ARG227	0.1322
THR233	1.5479	ALA228	0.8276
PHE234	0.9323	PHE229	2.3327
LEU248	0.2373	ALA230	2.4113
ILE249	1.8502	PHE231	3.3016
ILE250	1.0547	VAL232	2.8077
GLY252	0.1553	THR233	1.7541
ILE253	1.6086	PHE234	1.4693
SER254	0.9271	ILE250	0.9764
VAL255	0.228	ILE253	0.2795
		VAL255	0.7152

We analyzed how different truncations influence the solvent exposure of the NES residues (ILE239, LEU243, LEU248, and ILE250). As reported in Table 5, in fRRM2 these residues are mostly buried, with mean SASA values consistently below 0.5 Å^2^. Upon truncation, both tRRM2B and tRRM2A display conformations in which the NES residues become more solvent-exposed. The behavior of tRRM2A-l differs markedly, showing distinct features before and after the structural rearrangement associated with unfolding (Figures 4a,c). In the first half of the simulation, tRRM2A-l exhibits the highest degree of NES exposure, with mean SASA values exceeding 0.5 Å^2^ and reduced variability compared to tRRM2A and tRRM2B, suggesting a stable solvent-accessible configuration. In contrast, during the second half of the trajectory, partial refolding leads to a decrease in solvent exposure for residues 239 and 243, accompanied by higher fluctuation in SASA values. Overall, these findings highlight that variations in the structure and dynamics of truncated RRM2 fragments can strongly affect NES exposure and, consequently, their aggregation behavior. Since mutational studies have demonstrated that nuclear export requires the complete NES sequence (Winton et al., 2008), it can be hypothesized that stabilizing conformations in which certain NES subsets are buried might allow partial intervention in the pathological process. Such observations emphasize the importance of considering the specific conformational landscape of each fragment when designing molecules aimed at preventing TDP-43 aggregation and stabilizing non-pathological structural states.

**TABLE 5:** SASA of NES residues. For each MD simulation, the mean and standard deviation of the SASA of the NES residues are computed over all the trajectory frames. In the case of fragment tRRM2A-l, the SASA values were averaged separately over the two trajectory segments corresponding to the distinct conformational states observed before and after unfolding.

NES residue	fRRM2	tRRM2B	tRRM2A	tRRM2A-l I	tRRM2A-l II
239	(0.47 ± 0.12) Å^2^	(0.79 ± 0.42) Å^2^	(0.90 ± 0.40) Å^2^	(0.89 ± 0.13) Å^2^	(0.42 ± 0.19) Å^2^
243	(0.13 ± 0.09) Å^2^	(0.72 ± 0.52) Å^2^	(0.48 ± 0.42) Å^2^	(0.59 ± 0.14) Å^2^	(0.20 ± 0.11) Å^2^
248	(0.22 ± 0.11) Å^2^	(0.30 ± 0.19) Å^2^	(0.88 ± 0.26) Å^2^	(0.51 ± 0.12) Å^2^	(0.72 ± 0.27) Å^2^
250	(0.08 ± 0.11) Å^2^	(0.49 ± 0.14) Å^2^	(0.40 ± 0.35) Å^2^	(0.51 ± 0.14) Å^2^	(0.99 ± 0.17) Å^2^

## 3 Conclusion

Despite its relevance to a widely prevalent disease such as ALS, the molecular mechanisms underlying CTFs aggregation are still under investigation. In this study, we focused on the RRM2 portion of CTFs, which has been shown to play a critical role in aggregation (Johnson et al., 2008). By performing 10 μ s-long simulations of the full RRM2 domain (fRRM2) and its two fragments found in CTF aggregates (tRRM2A and tRRM2B), we quantified the structural and dynamic differences among them, showing how cleavage at different sites can induce distinct conformational changes and potentially lead to different aggregation pathways.

The developed protocol enables the prediction of aggregation propensity and possible aggregation path ways, while circumventing the limitation that aggregation events typically occur on timescales significantly longer than those accessible to unbiased simulations of this system size. Two models are commonly proposed to describe inclusion formation in neurodegenerative diseases: (i) amyloid fibrillization, in which highly insoluble aggregates with cross- β architecture are formed (Eisenberg and Jucker, 2012), and (ii) LLPS, where proteins demix into dynamic liquid-like droplets that can subsequently harden into amyloid-like assemblies under pathological conditions (Gomes and Shorter, 2019; Kato et al., 2012; Patel et al., 2015; Qamar et al., 2018). It has been hypothesized that TDP-43 CTFs aggregation may follow the amyloid model, driven by β -sheet stacking within RRM2 fragments, which have been found to aggregate even in the absence of the CTD (Wang et al., 2013). In this mechanism, adjacent β -strands form steric zippers, with side chains from neighboring sheets interdigitating roughly perpendicular to the fibril axis, generating the characteristic cross- β architecture observed in amyloid fibrils.

Our simulations show that RRM2 truncation leads to heterogeneous structural reorganization, loss of the stabilizing core of hydrophobic residues, and increased conformational sampling relative to the intact domain, consistent with previous experimental findings (Mackness et al., 2014). These cleavage-dependent structural changes seem to underlie distinct aggregation pathways. In tRRM2B, residues identified as aggregation-prone are often involved in β -sheets, suggesting that this fragment could follow the amyloid fibrillization model, in agreement with previous *in silico* studies (Grassmann et al., 2021). In contrast, tRRM2A exhibits even higher conformational variability, loss of secondary structure, and less frequent involvement of β -residues in aggregation-prone regions. This suggest that tRRM2A, which displays the highest predicted aggregation propensity, might follow a mechanism more consistent with LLPS-like aggregation.

To further explore tRRM2A and provide insights for potential therapeutic targeting, we analyzed an extended fragment, tRRM2A-l, which includes additional residues at the C-terminus, corresponding to an alternative RRM2 definition found in literature. An additional 10 μ s-long MD simulation revealed that tRRM2A-l undergoes distinct conformational dynamics, including transitions between folded and partially unfolded states. While these structural variations do not substantially alter the predicted aggregation propensity, they induce major conformational rearrangements relative to tRRM2A.

The structural and dynamic variations in truncated RRM2 fragments strongly affect NES accessibility. Notably, one of the two conformations sampled by tRRM2A-l exhibits the highest NES exposure, making it the most likely contributor to this aspect of the pathological process.

Overall, these results suggest that the precise length and dynamics of RRM2 fragments critically influence early misfolding events and should be considered when designing strategies aimed at preventing TDP-43 aggregation.

## 4 Materials and methods

### 4.1 Structural data

The RRM2 domain was extracted starting from the Nuclear Magnetic Resonance (NMR) structure of the TDP-43 tandem RRMs in complex with UG-rich RNA available on the Protein Data Bank (Bernstein et al., 1977) (PDB id: 4BS2) (Lukavsky et al., 2013). To obtain the fRRM2 fragment we removed both the RNA and the RRM1 domain. The resulting structure contains residues from 192 to 261 of TDP-43. Next, to obtain the molecular structure of fragment tRRM2B, we removed residues up to the 208th, whereas, to obtain fragment tRRM2A, we removed the residues up to the 219th.

For fragment tRRM2A-l, we manually selected residues from 220 to 269 from the PDB id: 4BS2, according to another standard definition of RRM2 found in literature (Lukavsky et al., 2013; Garnier et al., 2017; Mollasalehi et al., 2020).

### 4.2 Molecular dynamics simulations

All simulations were performed using Gromacs (Van Der Spoel et al., 2005). Topologies of the system were built using the CHARMM-36 force field (Best et al., 2012). Each system was placed in a dodecahedric simulative box, with periodic boundary conditions, filled with TIP3P water molecules (Jorgensen et al., 1983). For all simulated systems, we ensured that each atom of the proteins was at least 1.1 nm from the box borders. Each system was then minimized using the steepest descent algorithm. Next, a relaxation of water molecules and thermalization of the system were performed in NVT and NPT ensembles, each for 0.1 ns at 2 fs time-step. The temperature was maintained at 300 K with v-rescale thermostat (Bussi et al., 2007); the final pressure was fixed at 1 bar using the Parrinello-Rahman barostat (Parrinello and Rahman, 1980). The LINCS algorithm (Hess et al., 1997) was applied to constrain bonds involving hydrogen atoms. A cut-off of 12  Åwas used for the evaluation of short-range non-bonded interactions, and the Particle Mesh Ewald method (III Cheatham et al., 1995) was applied for the long-range electrostatic interactions. This procedure was followed for all the simulations.

### 4.3 Statistics and Reproducibility

All MD simulations were run for 10 μ s, ensuring that each system reached equilibrium conformations. All subsequent analyses were performed by removing the first 50 ns of the simulations to focus on the equilibrium range.

Several key structural and dynamical parameters (such as RMSD and radius of gyration - see Figure 1) were monitored to check if they reached stable values over the course of the simulations.

The length of the simulations performed guarantees that the protein fragments are sufficiently sampling the dominant conformational states.

### 4.4 Principal Component Analysis

PCA is a multivariate statistical technique used to reduce the degrees of freedom in a dataset. The original basis vectors describing the data is transformed in an orthogonal basis formed by the eigenvectors (also known as PCs) of the covariance matrix $\hat{C}$ associated with the observables. Each eigenvector is associated to an eigenvalue, indicating the amount of variance it captures from the data. The PCs can be ranked in descending order based on their eigenvalues; by selecting the first d PCs, the dimensionality of the dataset can be reduced while preserving its essential information.

### 4.5 Clustering analysis and representative conformations selection

To find the most representative conformations of a trajectory projection on its first two PCs, we implemented the k-means clustering algorithm. This algorithm is often implemented to reduce the dimensionality of MD simulation trajectories data, by decreasing the number of structures to be analyzed while preserving essential structural and dynamical information. MD conformations are grouped in clusters of similar structures according to their projection location in the PCs space. We then select the centroid of each cluster as the representative conformation for that class of structures.

To identify the best number of clusters (i.e., the value of k ), the k-means clustering maximizes the Silhouette Coefficient (SC), which quantifies how well a data point fits into its assigned cluster. For each point i in a cluster $C_{i}$ , it computes the silhouette value, as defined in Equation 1:

$$s\left(i\right)=\left\{\begin{array}{cc} \frac{b\left(i\right)-a\left(i\right)}{max\left(a\left(i\right),b\left(i\right)\right)}, & \text{if }|C_{i}|>1\\ 0, & \text{if }|C_{i}|=1, \end{array}\right.$$ (1)

where a(i) is called similarity and is defined, as shown in Equation 2:

$$a\left(i\right)=\frac{1}{|C_{i}|-1}\underset{\underset{i\neq j}{j\in C_{i}}}{\sum }d\left(i,j\right),$$ (2)

with d(i,j) the distance between data points $x_{i}$ and $x_{j}$ in the cluster $C_{i}$ . b(i) is the dissimilarity and is defined as in Equation 3:

$$b\left(i\right)=\underset{k\neq i}{min}\underset{j\in C_{k}}{\sum }d\left(i,j\right).$$ (3)

s(i) ranges between − 1 and 1; a value near one indicates that the point has been clustered appropriately. The mean s(i) over all data of the entire dataset, $\tilde{s}$ , measures of how appropriately the data have been clustered. The maximum value of the mean over all data of the entire dataset is the SC.

## Data Availability

The data that support the findings of this study are available from the corresponding author upon request.
